# Intranasal Lobular Capillary Hemangioma with Multiple Sites of Origin during Pregnancy: A Case Report and Literature Review

**DOI:** 10.1155/2018/7413918

**Published:** 2018-09-18

**Authors:** Lama S. Alalula, Abdullah S. Arafat, Riyadh A. Alhedaithy, Mohammed Elkrim

**Affiliations:** ^1^College of Medicine–King Saud bin Abdulaziz University for Health Sciences, Riyadh 11426, Saudi Arabia; ^2^Division of Otolaryngology–Head and Neck Surgery, Department of Surgery, King Abdulaziz Medical City, National Guard Health Affairs, Riyadh, Saudi Arabia

## Abstract

In the present case report, we describe a 33-year-old pregnant woman in the third trimester with a history of recurrent epistaxis leading to frequent visits to the emergency department. Each episode of epistaxis was managed by anterior nasal packing. During endoscopic examination, a left nasal mass was seen. She was admitted and managed conservatively until she delivered her baby without complication. After delivery, a CT scan was taken, which showed an enhancing mass in the middle and lower meatus of the nasal cavity with no bony erosions. For symptomatic relief and tissue diagnosis, endoscopic surgical removal was advised. An intraoperative examination revealed a red, smooth, and well-circumscribed mass occupying the left nasal cavity and originating from the medial surface of the inferior turbinate and the inferior surface of the posterior part of the middle turbinate. A complete en bloc resection of the mass was performed endoscopically. The mass was sent for histologic analysis, which confirmed the diagnosis of lobular capillary hemangioma. Eventually, upon follow-up at two weeks, one month, three months, and six months postsurgery, no evidence of recurrence was detected.

## 1. Introduction

Hemangiomas are benign vascular lesions commonly found on the skin and oral mucosa, with the nasal cavity being a rare site of involvement [1, 2]. They are classified according to their histopathological features. Lobular capillary hemangiomas (LCHs) visualized with a microscope contain numerous lobulated and dilated capillaries [1, 3]. Many names have been given to LCHs, including pyogenic granuloma and capillary granuloma, which indicate that its pathophysiology is still unknown [1, 4]. Studies have shown LCHs development to be associated with hormonal factors and local traumatic events to the skin or mucosa [1–5]. The anterior septum is the most frequently affected site, with epistaxis and nasal obstruction being predominating symptoms [1–5]. Proper investigations, including those that employ imaging techniques such as nasal endoscopy, are essential to differentiate LCHs from other dangerous entities, such as inflammatory or neoplastic lesions [1–5]. Despite the availability of multiple treatment options, surgical excision is the most effective form of management [1, 2]. In this case report, we describe a 33-year-old female patient in late pregnancy with a history of recurrent episodes of epistaxis and multiple visits to the emergency department. She was also found to have LCH with unusual and multiple sites of origin in the lateral wall of the nasal cavity.

## 2. Case Presentation

**Figure 1:**
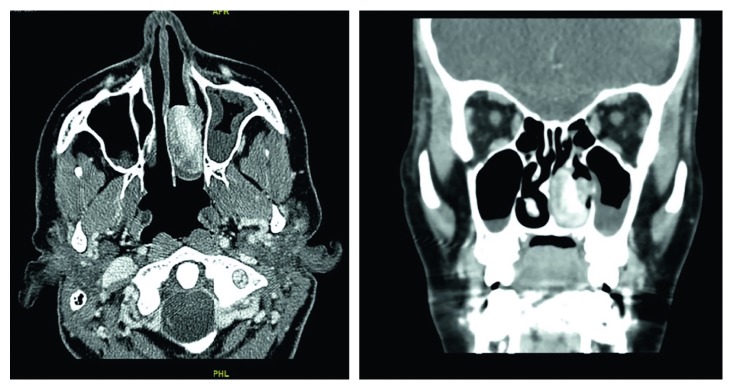
Axial and coronal sinus CT scans with contrast showing a heterogeneously enhanced mass involving the middle and lower meatus of the left nasal cavity with normal adjacent bony structures. In its maximum dimensions, the mass measured 3.2 × 2.2 × 1.5 cm.

**Figure 2:**
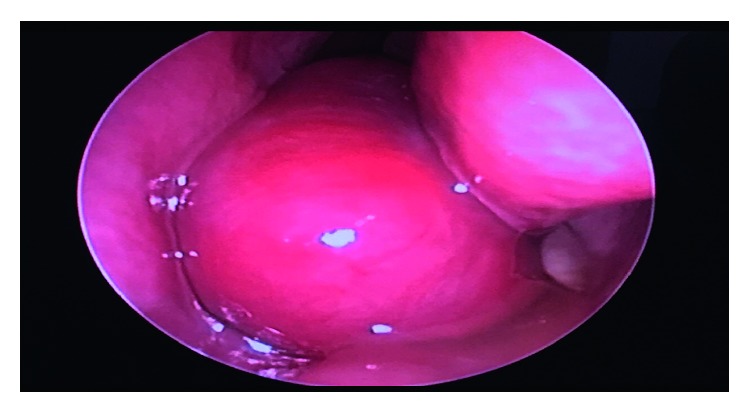
A preoperative endoscopic examination revealed a mass occupying the left nasal cavity.

**Figure 3:**
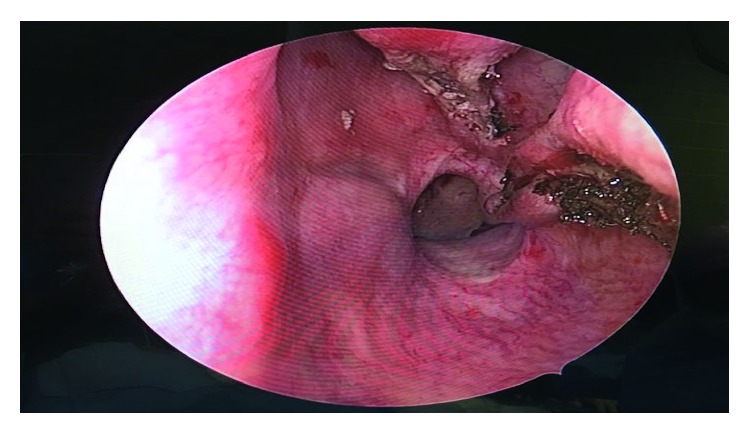
An intraoperative endoscopic examination after complete excision of a left nasal cavity mass revealed two sites of origin in the lateral nasal wall: the medial surface of the inferior turbinate and the inferior surface of the posterior part of the middle turbinate.

**Figure 4:**
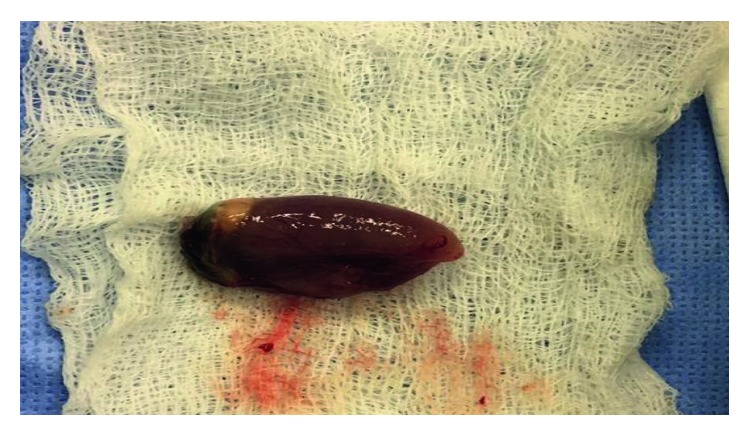
Gross appearance of the mass after excision, which had a smooth and glistening surface.

**Figure 5:**
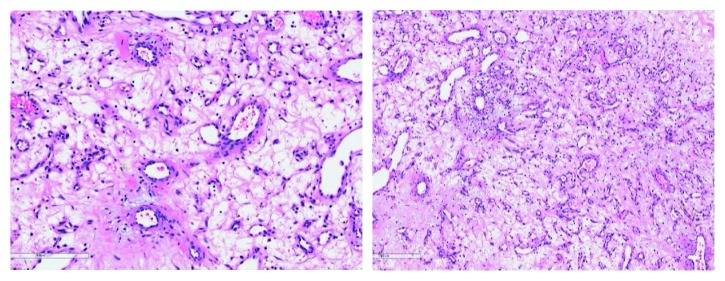
Microscopic examination of the tumor revealed lobulated and dilated capillaries with edematous stroma, confirming the diagnosis of lobular capillary hemangioma.

**Figure 6:**
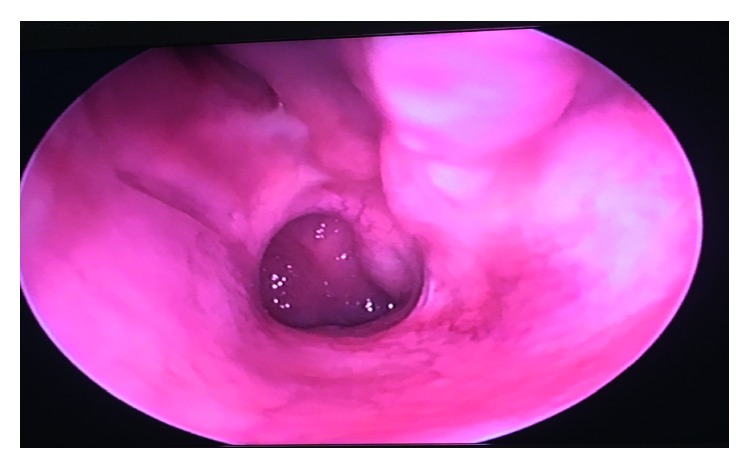
A 3-month postoperative examination via endoscopy indicating good mucosal healing at the site of procedure with no evidence of recurrence.

In June 2017, a 33-year-old gravida 3, para 2 female in her third trimester presented (ED) with active left nasal bleeding to the emergency department at King Abdulaziz Medical City in Riyadh, Saudi Arabia. The patient's vitals were stable upon presentation and she denied any history of trauma or nose picking. Additionally, she complained of a one-month history of persistent left-sided nasal obstruction. The patient was free of medical diagnoses and had no personal or family history of bleeding disorders or any other conditions. Anterior nasal packing was applied, and bleeding stopped two hours later. Her hemoglobin level was 9.9 mg/dL. Normal saline nasal irrigation was prescribed, and first aid instructions were given. Nasal packs were removed, and the patient was advised to come back if bleeding recurred. Ten days later, the patient returned to the ED with another episode of epistaxis that was managed conservatively. At that time, her hemoglobin levels were 9.4 mg/dL. She was discharged and advised to follow up with otorhinolaryngology. On the same day, she arrived at the ED for a second time with epistaxis of moderate severity. Again, minimal anterior nasal packing was applied, and the patient was sent home. On the next day, she returned to the ED for the third time in 48 hours with active bleeding from her left nostril. Her hemoglobin level at this point measured 8.7 mg/dL. During a bed side examination, the right nasal cavity appeared clear. However, the left nasal cavity evidenced a large clot with moderate bleeding. A nasal endoscopy was performed, which revealed a large, red, smooth, and rounded mass in the left nasal cavity that was actively bleeding upon any application of pressure. The bleeding stopped with properly sized anterior nasal packing, and the patient was rehydrated with intravenous fluids. Obstetrics and Gynecology and Otorhinolaryngology (ORL) teams were consulted. Since the patient was in her 38th week of pregnancy, a decision was made to retain the nasal pack for 48 hours more and admit her for spontaneous delivery, after which she would be reassessed. Two days later, the patient delivered, and both the mother and the baby were in good health. The day after delivering, the patient was taken to the ORL clinic for reassessment. The nasal pack was removed, after which the patient proceeded to actively bleed. Endoscopic assessment illustrated no changes to the nasal mass. Her hemoglobin dropped to 7.8 mg/dL. A blood transfusion was recommended, but the patient refused one. On the same day, a contrasted CT scan of the paranasal sinuses was scheduled, which revealed a heterogeneously enhanced soft tissue mass involving the middle and lower meatus of the left nasal cavity measuring 3.2 × 2.2 × 1.5 cm with normal adjacent bony structures (Figure 1). Two treatment options were discussed with the patient. The first was to wait for spontaneous regression of the mass following hormonal withdrawal after delivery. The second was to undergo complete surgical excision for quick symptomatic relief and tissue diagnosis, which the patient agreed to. The surgery was explained to the patient and informed, written consent was obtained. During intraoperative examination using a telescope, a large, well-circumscribed, red, smooth mass occupying the left nasal cavity and originating from the medial surface of the inferior turbinate and the inferior surface of the posterior part of the middle turbinate was visualized without any attachment to the nasal septum (Figure 2). The mass was initially injected with lidocaine and epinephrine (1 : 100,000) and then excised completely from its site of origin via bipolar diathermy which resulted in no significant bleeding (Figure 3). Gross histopathological analysis revealed a polypoidal tissue mass measuring 3.0 × 1.5 × 1.0 cm with a smooth and glistening surface (Figure 4). Microscopically, the mass was composed of numerous capillaries, likely associated with edema, and inflamed stroma with no malignant cells noted. Based on this, a diagnosis of LCH was made (Figure 5). Postoperatively, left-sided nasal obstruction markedly improved. The patient was stable and recovered well with no additional episodes of epistaxis or requirement for blood transfusion. She was consequently discharged on the same day in good condition. The patient was completely asymptomatic during follow-up appointments in our clinic at two weeks, one month, three months, and six months postoperatively, with healthy mucosa and no evidence of mass recurrence during endoscopic nasal examination (Figure 6).

## 3. Discussion

Lobular capillary hemangioma is a rare, rapidly growing, benign tumor featuring vascular malformation [1, 5]. It was first described by Poncet and Dor in 1897 as botryomycosis hominis based on the assumption that it arose secondary to a fungal infection [6]. Because it has since been shown to be neither infectious nor granulomatous, the term pyogenic granuloma is now considered a misnomer [2, 3]. Based on its histopathological features, the name LCH was assigned to these tumors by Miller [7]. Grossly, it appears as a polypoidal mass with an ulcerated surface that is covered with fibrin and blood [7]. Microscopically, it is further characterized by an association with numerous capillary sized blood vessels featuring edematous and inflamed stroma [2–4, 7]. LCH can be either pedunculated, meaning that it has a stalk that attaches the mass to its site of origin, or it can be sessile, meaning that the mass is fixed to its base without any peduncles [8]. Mills et al. reviewed 639 vascular lesions found in the oral cavity and upper respiratory tract [7]. Of those lesions, 73 had characteristics of LCH. Among those cases, the most commonly affected site was the lip, followed by the nose, oral mucosa, and tongue. Another study by Ash and Old reviewed 3000 nasal polyps, 23 of which were found to be sinonasal hemangiomas [9]. When found in the nasal cavity, the most common site for these tumors to present is on the anterior nasal septum, followed by the nasal vestibule, inferior turbinate, middle turbinate, and uncinate process, as Puxeddu et al. concluded in a retrospective study of 40 patients [4, 10]. In pregnancy, the prevalence of these tumors ranges from 0.5% to 5% [10].  Table 1 summarizes five different cases of pregnant women in whom LCHs originated from dissimilar sites and were of different sizes [11–15]. LCH affects both genders beyond the age of 40 years equally. Despite this, there is a predominance of females who are affected by LCH in the third decade while males are more often affected if less than 18 years old [16]. At present, no underlying mechanisms for the development of LCH have been demonstrated. Nevertheless, the most prevalent risk factors for LCH development include local trauma to the skin and mucus membranes, as is seen with nose picking, as well as hormonal imbalances, as occur in pregnancy and in women who use oral contraceptives or are postmenopausal [16]. Furthermore, LCH often presents with recurrent unilateral epistaxis, nasal obstruction, and discharge, and proper examination and imaging studies are essential to exclude other causes of a similar intranasal mass, inflammation, or neoplastic lesions [1]. The lobular arrangement of nasal capillaries differentiates LCH from granulation tissue in which there is a parallel capillary arrangement. Telangiectasia should be excluded if the patient has a family history of hereditary hemorrhagic telangiectasia and angiofibroma should be considered in the differential diagnosis if the patient is a male adolescent [1]. Typically, LCH presents as a well-circumscribed mass with marked enhancement and an absence of calcification on CT scans [17]. In the literature, different modalities have been utilized to treat LCH, such as intranasal steroids and harmonic scalpel, though endoscopic surgical resection remains the treatment of choice [5].

## 4. Conclusion

Based on the case study presented here, lobular capillary hemangioma (LCH) should be suspected if a pregnant woman has an intranasal mass causing nasal obstruction and recurrent epistaxis. In the patient described here, pregnancy was a risk factor that might have contributed to the development of LCH. However, further studies are needed to demonstrate the cause-effect relationship between pregnancy and LCH. Furthermore, the mass originated from the inferior and middle turbinate, which are uncommon sites of involvement. Finally, this patient underwent endoscopic surgical excision, which is the treatment of choice most supported by the literature, and was given a histologic diagnosis of LCH.

## Figures and Tables

**Table 1:** A summary of the case presented in this report and other lobular capillary hemangioma cases found in the literature.

Case	Age	Trimester	Symptoms	Size	Site	Treatment
Our patient	33	Third	Epistaxis and nasal obstruction	3.2 × 2.2 × 1.5 cm	The medial surface of the inferior turbinate and the inferior surface of the posterior part of the middle turbinate	Postpartum excision under general anesthesia
1	35	Third	Epistaxis and nasal obstruction	1 × 1 cm and a pedicle with a base of 0.5 cm	Anterior part of the septum in the right nasal cavity	Antepartum excision under local anesthesia
2	33	Third	Nasal obstruction	2 × 1.5 cm	Lateral nasal wall in the nasal valve area of the left nasal cavity	Postpartum excision under local anesthesia
3	34	Third	Epistaxis and a rapidly growing mass	25 × 32 × 16 mm	Attached to the nasal septum	Antepartum excision under general anesthesia
4	34	After delivery	Epistaxis and nasal obstruction	4 × 5 cm	Inferior turbinate in the left nasal cavity	Preoperative embolization and postpartum excision under general anesthesia
5	30	Third	Epistaxis and nasal obstruction	4.0 × 2.8 × 2.0 cm	Right nasal vestibule	Postpartum excision under general anesthesia
